# Integration of Technologies for Advanced Monitoring and Improved Performance of Filtration/Photocatalysis Systems in Water Treatment

**DOI:** 10.1021/acsomega.6c00220

**Published:** 2026-04-10

**Authors:** Hernán Dario Rojas-Mantilla, Juliano Passaretti Filho, Saidy Cristina Ayala-Durán, Maria Valnice Boldrin Zanoni

**Affiliations:** São Paulo State University (UNESP), Institute of Chemistry, Department of Analytical Chemistry, National Institute of Alternative Technologies for the Detection, Toxicological Evaluation and Removal of Micropollutants and Radioactivies (INCT-DATREM), Rua Professor Francisco Degni, 55, Araraquara - 14800-060, São Paulo State, Brazil

## Abstract

This study describes the development and evaluation of an economical, portable online monitoring system (OMSys) based on ESP32 Wi-Fi/Bluetooth technology, commercial sensors, and electronic measurement modules. The OMSys was designed for real-time evaluation of parameters such as the flow rate, temperature, and pH in a filtration/photocatalysis reactor used to degrade contaminants in treated water. The developed system allows simultaneous acquisition of all studied parameters at 2 s intervals and facilitates real-time data transmission via Wi-Fi, automatic storage, and real-time control (on/off) of other devices connected to the reactor, such as an LED irradiation source and a peristaltic pump, via a smartphone. The OMSys was evaluated in the degradation of venlafaxine (VEN) and used as a test model. Thanks to its performance, the OMSys facilitates the study of parameters that directly influence the degradation of emerging contaminants in real time, identifying critical stages in the water treatment process such as membrane stability (leaching). This enables real-time adjustments and improves the study’s final performance, demonstrating an approximately 80% reduction in VEN concentration within 120 min, a response influenced by changes in pH and temperature throughout the experiment. This system can be integrated with other recirculating loops for a variety of applications. The versatility of the OMSys is also evident, as it allows the addition of new sensors to monitor other parameters of interest. Easy upgrades with the addition of modern components improve performance, reduce problems such as obsolescence, and minimize environmental impact.

## Graphical abstract

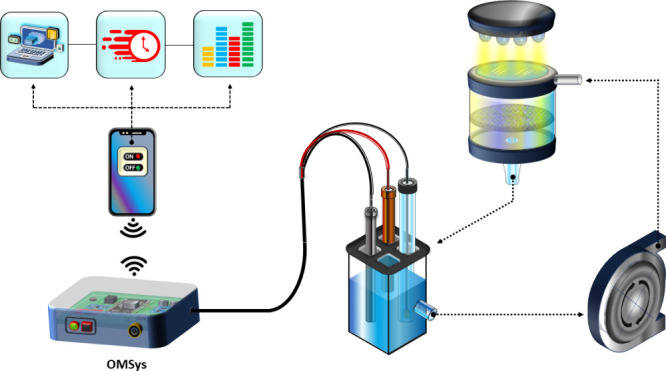

## 1 Introduction

The implementation of Industry 4.0 technologies is transforming conventional industrial processes through the use of new tools such as digitization, automation, and interconnectivity.^1^ This new approach facilitates real-time data communication and efficient information exchange between devices as well as improving fundamental aspects of industrial processes, such as detailed step tracking and rapid decision-making. In this context, online monitoring systems have shown significant growth due to their outstanding efficiency, ease of adaptation, and application, with an estimated growth of 29 billion devices in 2027 alone,^2^ impacting strategic sectors of society such as health,^3^ agriculture,^4^ energy,^5^ environmental monitoring,^6,7^ sensors,^8^ and, more recently, in photocatalysis,^9^ an area still little explored, as evidenced by the limited number of publications in the scientific literature. Another advantage of online monitoring systems is their ease of integration with commercial equipment, such as reactors and electrochemical cells, thereby aiding the optimization and automation of chemical or industrial processes. Advances in electronic miniaturization also enable the construction of portable and lower-cost online monitoring devices, maintaining the sensitivity and reproducibility of expensive commercial equipment but making it accessible to universities or small research centers, constituting a significant step in the democratization of knowledge and production at various scales.^10^

In hybrid processes, such as filtration/photocatalysis, in situ monitoring of critical parameters like flow rate, temperature, or pH is essential, as it allows for understanding the process dynamics and to warn of the emergence of unwanted problems (membrane rupture, fouling, leaching, temperature variation, and pH, among others). It is important to note that pH changes are associated with analyte ionization in the reaction and can also alter the surface charges of membranes and catalysts. On the other hand, temperature increases permeability and directly affects the speed of photocatalytic reactions.^11,12^ Automation, in turn, reduces data collection times, operator dependence, decreases operational costs, and expands analytical capacity,^13^ making its application interesting in areas such as water quality assessment, effluent analysis, and particularly in contaminant degradation studies.^14^

Programmable electronic devices using the ESP32 microcontroller stand out as an alternative in the creation of portable devices due to their features, such as integrated connectivity (Wi-Fi and Bluetooth), low operating consumption (3.0 to 3.3 V), high processing capacity (dual-core 32-bit and 240 MHz), and large memory capacity (up to 512 KB), in addition to having support for external memories (Flash, PSRAM). As they have multiple digital inputs/outputs (34 pins) and support protocols such as I2C, SPI, and UART, they enable integration with various sensors and peripherals. These features allow for fast signal processing and increase the ability to perform tasks simultaneously. Communication with other devices, such as smartphones, tablets, or computers, is facilitated, and hardware encryption (AES, RSA, and SHA) is supported, ensuring data protection when necessary. Its low cost, easy programming due to compatibility with software such as the Arduino IDE or PlatformIO, an active community, and free access to libraries, tutorials, and other learning resources make it an ideal prototyping platform for the development of portable devices.^4,15^

On the other hand, the use of light-emitting diodes (LEDs) as efficient irradiation systems in processes such as photocatalysis is considered a promising alternative applied in the treatment of contaminated waters.^16,17^ Among their main advantages, the high energy efficiency shown (80–90%), luminous stability, long service life (approximately 50,000 h), and immediate on–off response stand out. In addition, they are produced across a wide spectral range (UV–vis–IR), with specific emission wavelengths, thereby enhancing catalyst activation and, consequently, the efficiency of photocatalytic processes.^18^ Compared with traditional sources such as mercury or xenon UV lamps, LED devices offer low heat emission, lower cost, and easier disposal. In addition, their compact form enables numerous arrangements, favoring miniaturization and compatibility with other equipment. The compatibility of these electronic components enables the development of smart lamps with automatic on/off switching and specific light-intensity settings, features that improve and further optimize their use in the treatment and purification of contaminated water.^19,20^

Several configurations and portable systems for the simultaneous monitoring of physicochemical parameters relevant to water quality have been reported in the literature.^6,21–23^ However, few studies have reported their use in photocatalysis or as monitoring systems for hybrid filtration/photocatalysis processes, which represent a very interesting scenario to investigate. In this regard, Qin et al.^24^ developed a portable, low-cost device that enables integrated monitoring of pH, temperature, and free chlorine concentration for evaluating water quality. The pH and temperature sensors were manufactured by simply using jet printing. The chlorine sensor was developed using an electrochemically modified graphite electrode and integrated into a FPGA (Field-Programmable Gate Array) board that served as the microcontroller. The device achieved high sensitivity, with values of 60.6 3.35, and 342 nA/ppm for pH, Temperature, and free chlorine concentration, respectively. It also achieved 82% accuracy when evaluated in real samples: Tap water (Toronto), Lake water (Lake Ontario), and Swimming pool water (McMaster University). The system, being a portable, economical, and easy-to-build device, also enables future integration with other electronic sensors to evaluate parameters such as conductivity, dissolved oxygen, and heavy-metal detection, thereby simplifying conventional analytical methods and proving to be a sustainable alternative for monitoring water quality.

Another advantage of this technology is the low cost of upgrading or modernizing commercial equipment. In this context Kalamaras^6^ automated a pilot-scale anaerobic bioreactor (60 L) used in the production of biogas for energy production, from an ESP32 microcontroller, coupled with several commercial electronic sensors that allow simultaneous monitoring of the following parameters of interest: pH (using a GE 117-BNC electrode coupled to a GPHU014 MP-BNC transducer), temperature (using a DS18B220 sensor), redox potential (using a GE 175-BNC electrode coupled to a GRMU 2000 MP-BNC transducer), and ammonium (NH_3_) concentration (using an ammonium ion selective electrode, 3051 (ISE)). In addition, this system enabled the detection of biogas volume and the % methane (CH_4_) content generated by the process. The developed device showed high accuracy and stability in data collection and storage and demonstrated high sensitivity, detecting small drops in biogas production. On the other hand, parameters monitoring of the yielded temperature and pH deviations (0.15% and 1.7%, respectively), as well as a CH_4_ concentration variation of less than 6.0%, compared with more robust and expensive conventional analytical methods, such as HPLC. However, the measurements for ammonium ion showed variations with averages of 10% to 20% after 11 and 20 days, respectively. In general, the system proved to be effective for real-time monitoring of critical parameters in anaerobic processes, demonstrating its viability for assisting and optimizing parameters in industrial equipment. On the other hand, its capacity for in situ and postreaction analysis and data collection provides a broader view of the physicochemical conditions and the process’s constant changes, facilitating decision-making during the experiment and, in the long term, reducing energy and operating costs through improved process optimization.

Compatibility, easy communication, and fast data transfer with other latest-generation devices, such as smartphones, as well as data management via an application interface (app), are other advantages of its technology. In this regard, Sneineh & Shabaneh^25^ developed a hydroponic monitoring system for lettuce crops, using an ESP32 microcontroller connected to several electronic sensors: pH (PH-4502C), temperature (DS18B20), and total dissolved solids (TDS) (using a printed circuit board). The information from the sensors is collected using a “Blynk” application on the smartphone as an interface, which allows the monitoring of parameters, data storage, and automation of tasks, such as activating the water pump, checking the water level, and controlled release of nutrients, such as phosphoric acid and (K, N, and P), remotely. Proved to be a promising, accessible, economical, and easy-to-apply alternative for the agricultural sector, favoring the optimal growth of the evaluated crops due to better control and improved monitoring of parameters in hydroponic systems.

Therefore, the objective of this work was to develop and evaluate a portable and low-cost device, named “OMSys”, capable of real-time monitoring of parameters such as flow, temperature, and pH, in addition to enabling continuous data collection, transmission, and storage. For evaluation purposes, the device was integrated into a filtration/photocatalysis reactor operated in a recirculating loop, applied in the degradation of the drug venlafaxine (VEN), an antidepressant considered an emerging contaminant (CEC), due to its high recalcitrance and toxicity to marine organisms, as well as its wide environmental occurrence.^26^ OMSys is expected to facilitate the study of parameters that directly influence the in situ degradation of emerging contaminants, identify critical stages in the filtration/photocatalysis process, such as membrane stability (leaching), and serve as a promising technological solution for monitoring in contaminated water treatment systems.

## 2 Materials and Methods

### 2.1 Reagents

The antidepressants venlafaxine (VEN) (99.9% purity) and acetonitrile (HPLC grade) were purchased from Sigma-Aldrich, Germany. Carbon fiber (PWB-3) was acquired from Zoltek, USA. CH_2_O_2_ was purchased from J.T. Baker. HNO_3_ (65.0%), Na_2_WO_4_ (99.9%), and H_2_O_2_ (29.0%) were purchased from Synth and used as received. Milli-Q Water was used to prepare all of the solutions.

### 2.2 Modification of CF/WO_3_ Membrane for Chemical Electrodeposition

The carbon fiber PWB3 modification followed the methodology.^27^ Electrodeposition was carried out in a standard three-electrode cell. As the work electrode, CF (99.99%) purity was used. A two-dimensionally stable anode (ADE De Nora) was used as a counter electrode, and Ag/AgCl (4.0 M KCl) was used as a reference electrode. In the cell, 200 mL of Na_2_WO_4_ electrolyte solution was added (5.0 mM), H_2_O_2_: 0.075% (v/v), with prior adjustment to pH 1.4 (HNO_3_). A potential of −0.5 V was applied to the system for 1 h using a potentiostat/galvanostat (Autolab, PGSTAT 302). Finally, the membrane was washed with water, dried at 60 °C for 2 h, and subsequently subjected to heat treatment at 450 °C with a heating ramp of 2 °C min^–1^ for 2 h.^28^

The CF/WO_3_ membrane was previously characterized and is discussed in Rojas-Mantilla.^29^

### 2.3 Degradation Experiments

The sensors were tested simultaneously in a filtration/photocatalysis reactor operating in a recirculating loop, with degradation experiments and photolysis and adsorption controls being carried out, under the following conditions: pH 6.5, VEN concentration 1.0 mg L^–1^, CF/WO_3_ membrane, volume 150 mL, irradiation (365 nm -12 W), and room temperature.

Before the degradation experiments were started, the membrane sorption/desorption equilibrium was reached by recirculating the VEN solution in the dark for 30 min. Subsequently, the LED irradiation system is turned on, and aliquots are collected at 0, 10, 20, 40, 60, and 120 min; the VEN concentration is monitored by HPLC.

### 2.4 Chemical Analysis

VEN concentration was determined by high performance liquid chromatography (HPLC) using a Shimadzu LC-20AT with diode array detector (DAD) in reversed phase, column Kinetex 5 μM EVO C18, dimensions: 150 × 4.6 mm, and guard column Phenomenex (4.0 × 3.0 × 50 mm), using oven temperature at 40 °C, Flow rate: 1.0 mL min^–1^, injection volume: 50 μL and in the linear gradient method from a formic acid 0.1% (v/v)/acetonitrile, under the following conditions: with an acetonitrile concentration of 15% to 100% in the first 12 min. Followed by a stabilization step for up to 19 min.

The analytical curve for venlafaxine was obtained in a linear range from 2.0 mg L^–1^ to 0.1 mg L^–1^, with y = 91258x - 5074.7 and R^2^ = 0.9994. The limits of detection (LOD) and quantification (LOQ) were determined using the standard deviation of the intercept (σ) and the slope of the regression (b) according to international analytical validation guidelines. The limits were estimated using LOD = 3σ/b and LOQ = 10σ/b, yielding values of 0.10 and 0.30 mg L^–1^, respectively. A VEN concentration of 1.0 mg L^–1^ was selected as the working concentration for evaluating the OMSys monitoring system in the recirculating loop, as it lies in the central region of the linear range, ensuring adequate sensitivity, precision, and reliability in quantification.

The elemental analysis of tungsten (W) present in the solution (leaching), before and after the photocatalysis processes, was quantified by Inductively Coupled Plasma Optical Emission Spectrometry (ICP-OES) using a Thermo Fischer iCAP 6500 Duo instrument, with the SMWW 3120 B method (n = 3) and applying the following conditions: pumping speed 50 rpm, power 1.150 W and tungsten (W) emission line of 239.7 nm.

X-ray diffraction (XRD) patterns were obtained by using a Rigaku Smartlab SE diffractometer. Measurements were carried out in the 2θ scan axis (5–80°), rate of 5° min^−1^, in standard Goniometer configuration, operating at 40 kV and 20 mA, with Cu–Kα (1.54186 Å) in D/teX Ultra 250 detectors.

### 2.5 Determining UV LED Optical Specifications

The irradiation system’s emission wavelength was confirmed using a USB 4000 spectrometer (Ocean Optics, USA) connected to a P400–2 UV/vis optical fiber. Lumens flux was determined using a digital luxmeter, INSTRUTHERM, model LD-300. The irradiance was measured in the UV region (320–400 nm) by using a PMA radiometer 2100 (Solar Light Co., USA).

## 3 Results and Discussion

### 3.1 Construction of OMSys

The online monitoring system was designed using an ESP32 microcontroller, an economical device with low energy consumption (0–3.6 V and 240 mA) that integrates an Xtensa 32-bit LX6, with a standard frequency (160 MHz) and processing (600 DMIPS), in which a set of sensors were coupled: (A) Water Flow Sensor Model: YF-S201; (B) corresponds to an open channel created for monitoring another parameter of interest for future study; (C) Relay Module: 2-channel (5 V/10A) with Opto-couplers, in addition to one Solid state relay SSR 3–32VDC 40 A; (D) Max6675 Temperature reader module, K-type thermocouple (0 to 700 °C) and (E) pH Sensor with PH4502C Module + Electrode (Figure 1a). The ESP32 allows Wi-Fi connection and data transmission via Bluetooth, even establishing point of connection with routers, facilitating the development of applications with Web server interfaces for different types of approved clients, promoting the transfer of technologies for laboratory or industrial applications (Figure 1b), making it ideal for process automation.^30^ For its programming, the source code was written and uploaded using Arduino IDE, version 1.8.13. But details of the complete source code are available in Appendix A of the Supporting Information. The control program created was named “fotoreator.ino” and applied to the Windows operating system (Figure 1c), but it is compatible with other operating systems such as Mac OS X or Linux. In addition, the ESP32 is compatible with Arduino libraries, which allows the use of available shields and libraries. The final system that completed the OMSys system is shown in Figure 1d.

**Figure 1:**
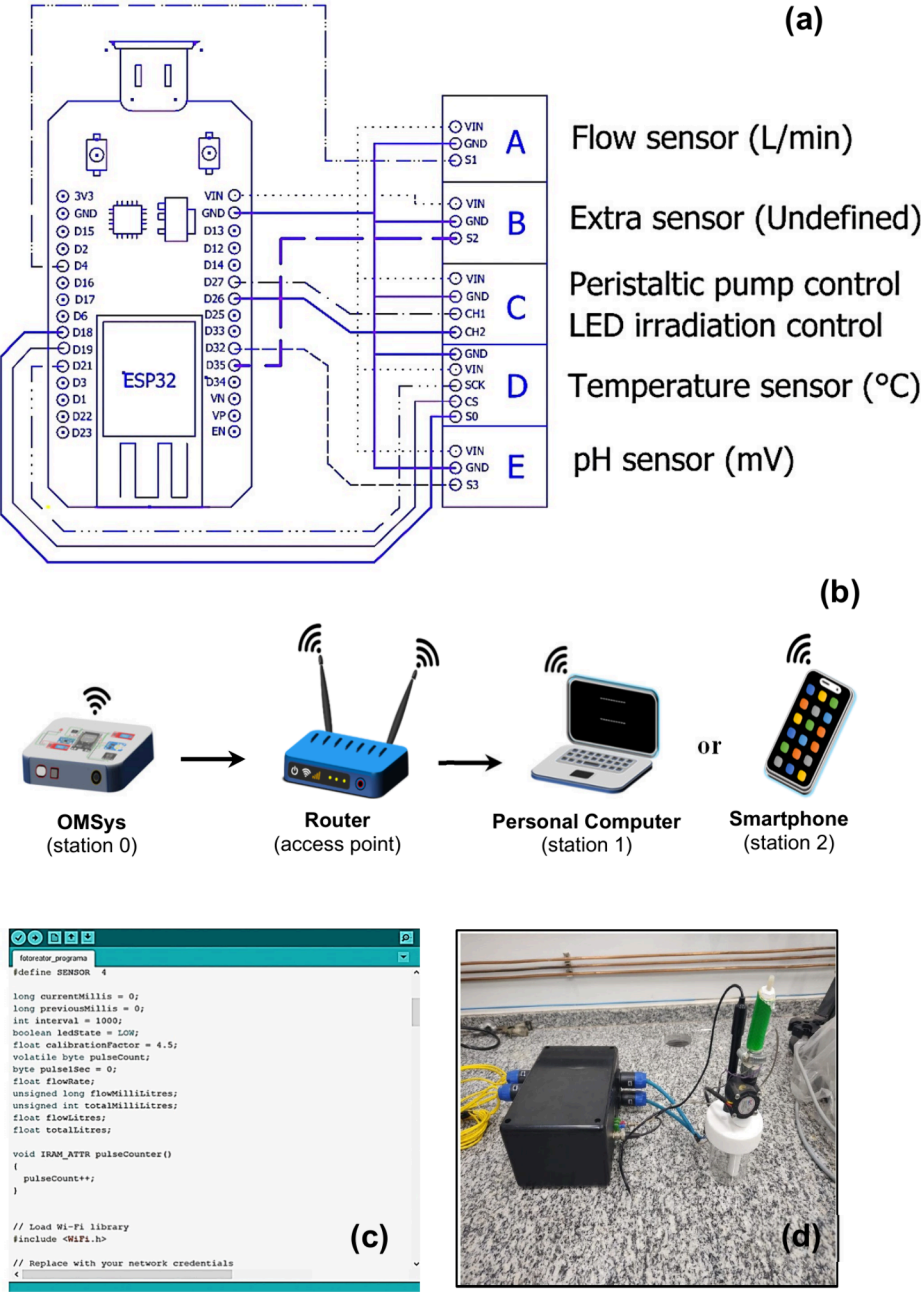
(a) Connection diagram of the ESP32 module with the sensors, (b) Wi-Fi communication from online monitoring system to computer or smartphone, (c) control program “fotoreator.ino” (Arduino software, version 1.8.13), (d) OMSys in operation.

The OMSys is controlled by the ESP32 microcontroller, transferring in real time information from a: (A) flowmeter with hall effect sensor, the program counts the revolutions of an internal fan present in the device; (C) 2 - Solid state relay (two channels), Power supply current (minimum): 160 mA and G3MB −202P relays used to turn on/off the irradiation system and peristaltic pump; (D) A K-type thermocouple using a MAX6675 chip, the chip performs cold-junction compensation and digitizes the signal of a K-type thermocouple. Data are output at 12-bit resolution in read-only format. This converter provides thermocouple accuracy of 8 LSBs over the temperature range 0 to 700 °C. (E) The pH Sensor, model PH4502C is a versatile and portable device showing a measurement range: 0 ∼ 14, works in a temperature range of 0–60 °C and alkaline error of 0.2 pH (Figure 2a). The entire set was mounted on an 8.0 × 12 cm printed circuit board (PCB) and protected by a 19 × 12 x 5 cm Project box. On the sides of the box, connections were made between the plates and the physical sensors, which were positioned vertically above a glass reservoir in the recirculating loop. (Figure 2b).

**Figure 2:**
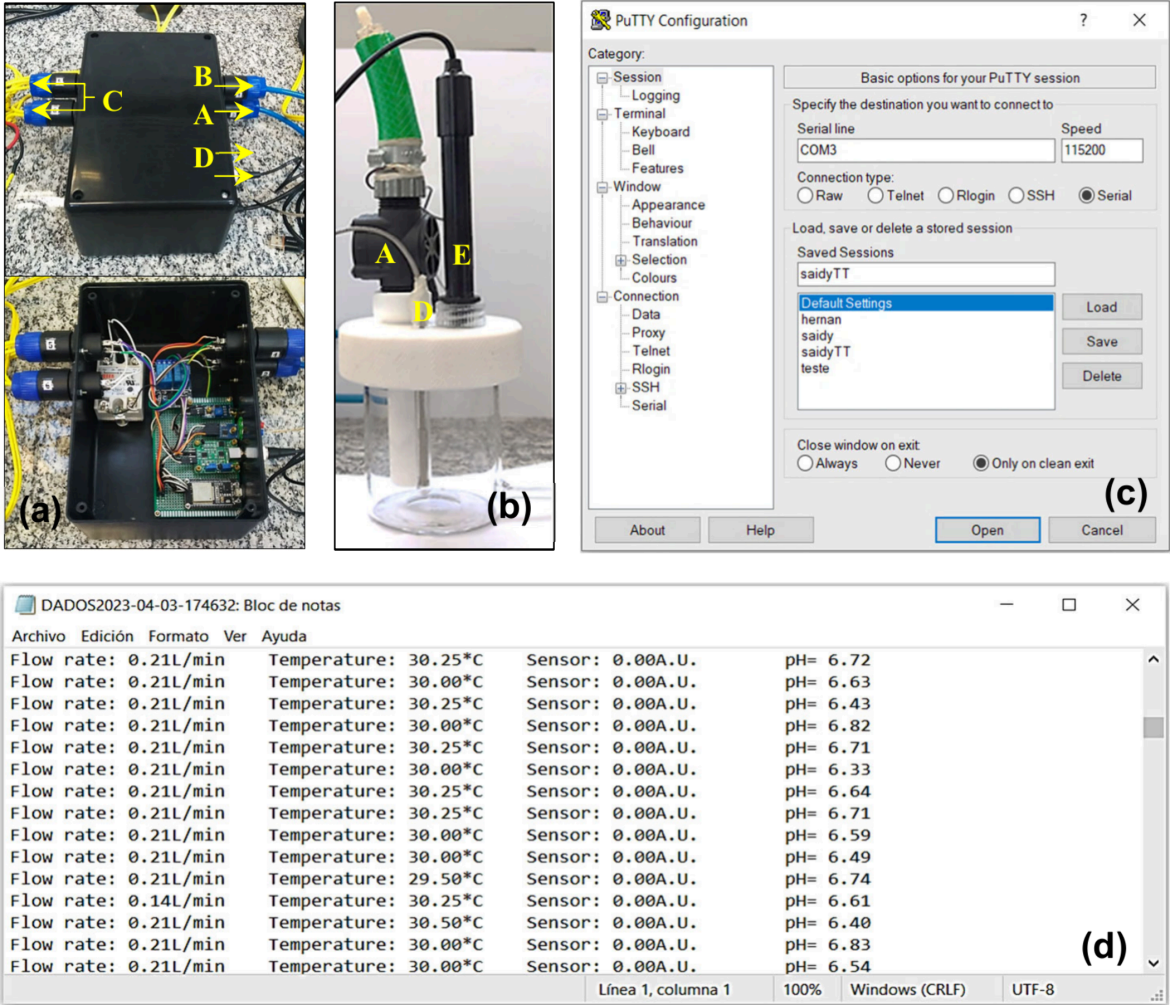
(a) Construction of the online monitoring system, (b) distribution of the sensors in the glass reservoir, (c) free and open-source PuTTY software, and (d) obtaining data (Notepad).

The information collected from each sensor (flow, temperature, and pH) is transmitted by the ESP32 to the computer via USB. Using the free and open-source software PuTTY (https://www.putty.org/), it is possible to monitor in real time the signals of sensors and states of relays (Figure 2c), as well as allowing the capture and recording of data in txt format and store them on the computer as a file (notepad) (Figure 2d). The developed online monitoring system showed adequate reproducibility, fast data acquisition (every 2.0 s), and automatic storage. In addition, it allows understanding of the current status of a process or reaction at any stage of the experiment. Another command, called “sensor” (Figure 2d), was included in the program and can be used to monitor another process parameter in the future.

For performance evaluation, the online monitoring system (I) was integrated with a filtration/photocatalysis process operating in a recirculating loop (Figure 3a), comprising a glass reservoir with a capacity of 150 mL, in which the flow, temperature, and pH sensors were submerged vertically (II). The solution inside the reservoir was driven through silicone hoses with the aid of a peristaltic pump (III) to a filtration/photocatalysis reactor (IV).^29^ This reactor has a side entrance, where the solution enters an internal chamber and passes through a photoactive CF/WO_3_ membrane; Simultaneously, the solution is irradiated through a quartz window located in the upper part of the reactor by using a UV-LED lamp (365 nm, 12 W) (V) and an adjusted power supply (VI). After passing through the membrane, the solution is directed perpendicular to the membrane and sent to the glass reservoir for recirculation until the end of the experiment.

**Figure 3:**
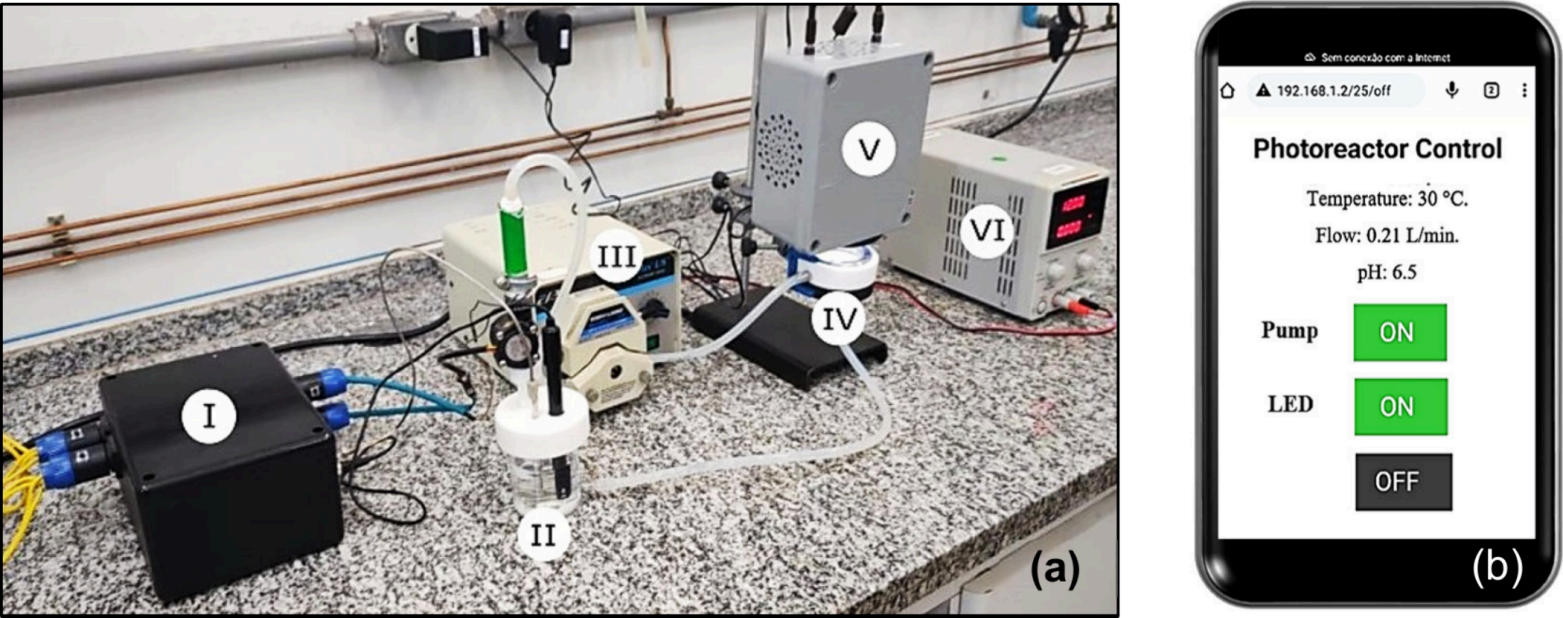
(a) OMSys in operation **(I),** glass reservoir with sensor **(II)**, peristaltic pump **(III)**, membrane reactor **(IV)**, UV-LED (365 nm) **(V),** and power supply **(VI)**; (b) on/off remote control of peristaltic pump and LED irradiation using smartphone.

The online monitoring system was also designed to remotely turn on/off the peristaltic pump and the LED irradiation system using devices such as smartphones or tablets (Figure 3b). This function is implemented via GPIO 26 and 27 of the ESP32 microcontroller.

### 3.2 Construction of the LED Irradiation System

The irradiation system was designed by using four UV LED chip devices (365 nm, 3 W) configured in a series circuit (Figure 4a). Thermal paste was applied to the base of the LEDs and then fixed to an aluminum heatsink (85 × 104 × 25 mm), after which their electronic interconnection was completed. To prevent overheating, a cooling fan measuring 80 × 80 × 25 mm was added to the back of the heatsink; it operates at 12 V and 0.5 A. The entire assembly was placed inside a fire-resistant Junction Box (170 × 145 × 90 mm, model Kraus Muller) and powered by a Power Supply 15 V/2 A, 30 W (Figure 4b). The emission spectrum of the LED irradiation system showed a peak at 365 nm, characteristic of the UV spectral band (Figure 4c). To optimize its operation, several ventilation outlets were added to the side walls of the box to allow for air circulation (Figure 4d). The developed irradiation system had the following optical specifications: luminous flux 13,000 lm, irradiance 58 mW cm^–2^, and average emission angle of 120°, these measurements were performed using a 2 cm optical path (Figure 4e). In this regard, it is known that the WO_3_ semiconductor exhibits broad absorption of radiation in the UV range (370–400 nm), making it ideal for photocatalytic processes.^31^

**Figure 4:**
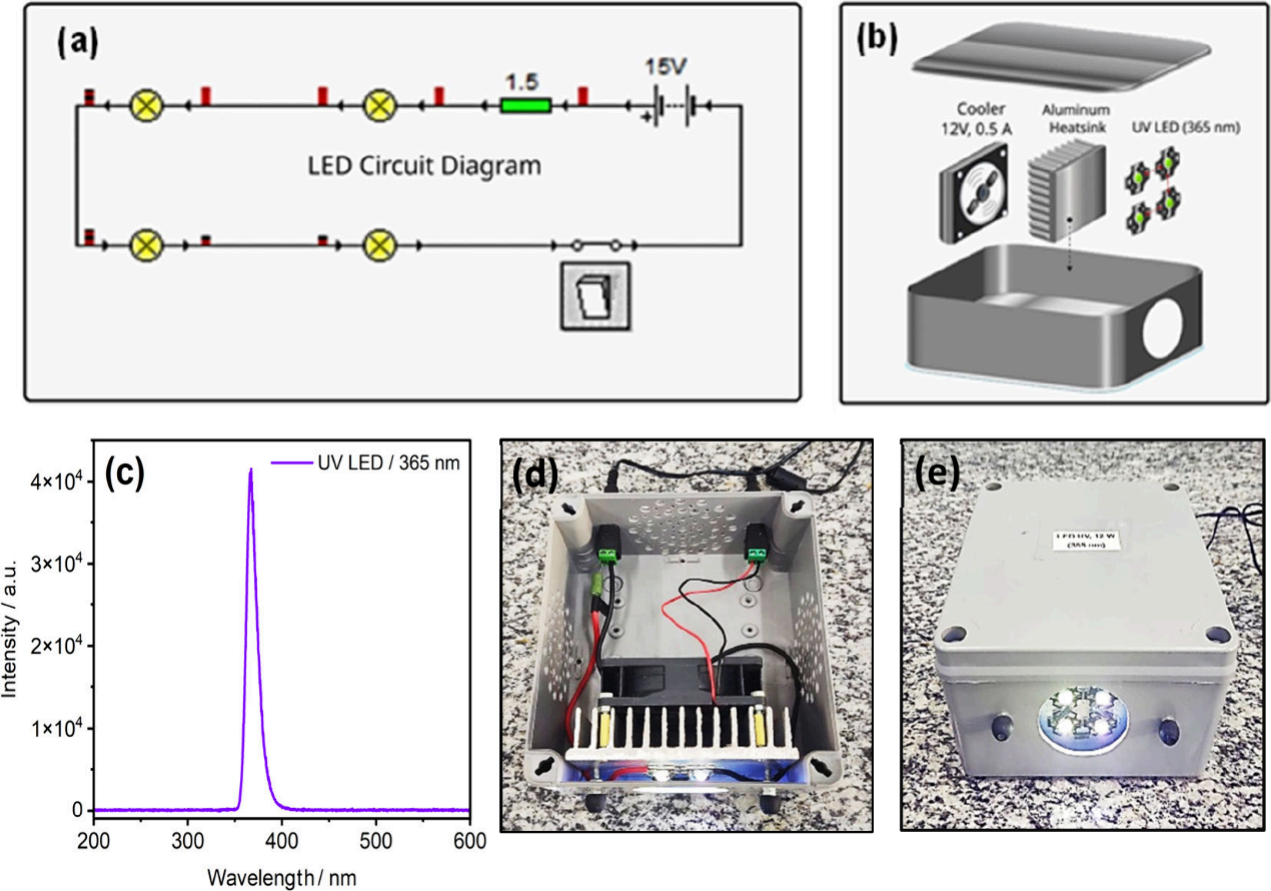
(a) Series circuit simulation performed with the free software Crocodile Clips 3.5. (b) UV LED system construction diagram. (c) Emission spectrum. (d) Internal view. (e) Irradiation system in operation.

### 3.3 Cost Estimate

A comparative analysis of the individual costs of the electronic components that make up the OMSys device and the LED irradiation system developed in this research (Table 1) was carried out and compared with the values of similar commercial devices to demonstrate their viability (Table 2). It is important to note that during the creation process, the proposed devices were designed to meet the same operating specifications as commercial devices, facilitating their future optimization. On the other hand, this type of alternative facilitates national scientific production by democratizing access to specialized knowledge, significantly reducing dependence on the acquisition of foreign equipment or technologies, which are characterized by high economic cost and limited accessibility, and encouraging high-quality national technological production.

**Table 1:** Production Cost Assessment of the OMSys Device and LED Irradiation System

OMSys		LED Irradiation system	
Component; (Quantity)	Price^a^(USD)	Component (Quantity)	Price^a^(USD)
ESP32 module - WiFi + Bluetooth; (1)	9.99	UV LED chip (365 nm, 3 W) (4)	19.99
Water Flow Sensor: YF-S201; (1)	9.49	Aluminum heatsink; (1)	3.99
Relay Module: 2-channel 5 V/10A); (1)	8.89	Brushless DC Cooling Fan 12 V/0.5A; (1)	8.99
Relay SSR 3-32VDC 40 A; (1)	12.29	Junction Box; (1)	14.99
Temperature module: Max6675; (1)	7.69	Plug Connector Female 12 V/5A; (2)	1.39
pH Sensor, PH4502C + Electrode; (1)	25.52	Power Supply 15 V/2 A, 30 W (1)	7.99
Project Box; (1)	8.99	**Total price**	**57.34**
Printed Circuit Board (PCB), 8 × 12 cm; (1)	3.07		
**Total price**	**85.93**		

a Commercial dollar price (10/11/2025) on Amazon.com.

**Table 2:** Comparison of OMSys and Irradiation LED System Device with Commercial Values

Commercial measuring equipment	Price^a^(USD)	Commercial LED irradiation system	Price^a^(USD)
Water Flow Control Meter LCD Display	64.99	UV LED lamp Thorlabs (365 nm, 6.8 W)	**506.06**
DTM-307 Digital Thermometer, k Type	60.00		
PH800 Laboratory Benchtop pH Meter	399.78		
**Total price**	**524.77**		

a Commercial dollar price (10/11/2025) on Amazon.

When analyzing the values shown in Tables 1 and 2, respectively, the cost of developing the OMSys devices and the LED irradiation system is six and nine times lower than those mentioned for equivalent commercial devices, evidencing the technical feasibility of the proposed devices, as well as their competitive potential in terms of cost and benefit. This is interesting, especially in contexts with limited financial resources.

### 3.4 Parameter Evaluation

Before applying OMSys to treat contaminated water, a thorough assessment of each sensor’s performance was conducted, considering sensor stability and the optimal data acquisition time. These tests were carried out under the same conditions that would be employed in the VEN degradation experiment using a filtration/photocatalysis photoreactor with CF/WO_3_ membranes operating in a recirculating loop; under the following conditions: pH 6.5; VEN concentration 1.0 mg L^–1^; volume 150 mL; LED irradiation (365 nm -12 W) and room temperature.

#### 3.4.1 Flow Sensor Evaluation

The stability and repeatability of the flow sensor response were evaluated for three different flow conditions, named respectively, low (140 mL min^–1^), medium (210 mL min^–1^) and high (360 mL min^–1^) (Figure 5). The results showed that, regardless of the flow used, the data set exhibits some variability, with changes occurring at specific time intervals, with averages of 143 ± 24, 207 ± 21 and 365 ± 25 mL min^–1^ for low, medium, and high flow, respectively.

**Figure 5:**
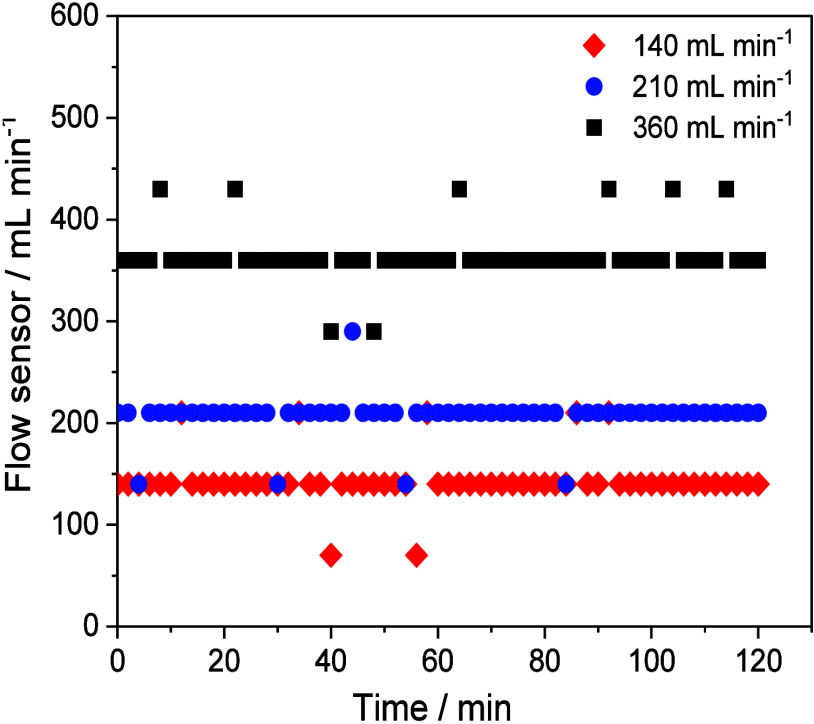
Performance and stability evaluation of the water flow sensor (Model: YF-S201) in a recirculating loop. *Experimental conditions:* CF/WO_3_ membranes; pH 6.5; VEN concentration: 1.0 mg L^–1^; volume 150 mL; LED (365 nm, 12 W) and variation in flow rate: 140, 210, and 360 mL min^–1^ (*n* = 1).

Some researchers report that variations observed in this type of sensor are expected.^32,33^ It is important to note that this type of sensor operates on the Hall effect, generating an electrical pulse for each rotor revolution. This generated electrical pulse can be affected by unwanted particles in the fluid, causing small oscillations in the measurements due to interference with the sensor’s magnetic field.^34^ On the other hand, as a general characteristic, this type of device commercially has an accuracy of ± 10%.^35^

When determining the relative standard deviation (%RSD) for each set of grouped data relative to the average, it was observed that the lowest flow used, i.e., 140 mL min^–1^, had a variation of 16% RSD. However, the medium and high flows, 210 and 360 mL min^–1^, showed RSDs of 10% and 7%, respectively, indicating that the sensor’s accuracy and repeatability are stable and lower at flows greater than 210 mL min^–1^.

All flow data were processed using a low-pass filter with a cutoff frequency of 0.0625 min^–1^ based on Fast Fourier Transform (FFT). When applying the FFT to the filtered signal, as shown in Figure S1, the filtered FFT signals showed that the flows remained practically constant around the calculated average value throughout the experiment, with some fluctuations that are attributed to experimental instabilities, pump pulsations, and mechanical vibrations that cause occasional small changes in outlier values.

Devices using Hall-effect flow sensors and Arduino have proven to be effective in other industrial applications. As reported,^36^ they developed a device that allows the generation, measurement, and image capture of microbubbles for the separation of oil from water samples, using an Arduino UNO R3 as a microcontroller, a Hall-effect liquid flow sensor YF-S201b coupled with microscope lenses and a GoPro camera. This system enabled precise measurement of the microbubble size and control of the minimum and maximum injected airflow limits. The system allowed monitoring of flow rates from 0.001 to 10,000 L h^–1^, with a linear regression coefficient of 98%.

Radj & Kamalanathan^37^ reported the use of a YFS201 Hall-effect sensor connected to an Arduino Uno microcontroller to develop a device that can be adapted to the outlet tube of gasoline dispenser guns, enabling high-precision measurement of the flow of gasoline supplied during vehicle refueling. The device proved to be effective and comparable to analogue fuel gauges.

McCarthy^38^ developed a low-cost peristaltic mini pump, by 3D printing, driven by a direct current motor, in addition to containing a hall sensor that allowed the detection of the pumping flow. The entire system was controlled using an Arduino Uno R3. This prototype achieved 99% accuracy in pumping volume, with flow rates up to 13 mL min^–1^, and demonstrated a pumping-volume uncertainty of only 0.7%, attributable to the use of a Hall-effect sensor.

#### 3.4.2 Temperature Sensor Evaluation

In processes involving photocatalytic membranes, the temperature is a critical parameter because it directly influences reaction kinetics and thermodynamics, making its monitoring essential. It is known that a determined increase in the process temperature has positive effects on photocatalytic performance, due to improved interfacial charge transfer, thereby providing better contact between the catalyst and the reaction medium.^39^ Although there is no total consensus, the ideal temperature range for photocatalytic processes is estimated to be between 25 and 45 °C.^39,40^ However, increases above 45 °C can lead to negative effects related to the rapid recombination of electron (e^–^)/hole (h^+^) pairs, as well as possible desorption of contaminants absorbed on the catalyst surface, resulting in low catalytic activity.^40^

When evaluating the performance of the digital thermometer (K-29) versus the Arduino Max6675 sensor, it was observed that both devices showed similar behavior according to the profile observed in Figure 6, reaching average temperatures of 27.3 ± 0.9 and 30.4 ± 1.4 °C with relative standard deviation (%RSD) of 4.5 and 3.2%, respectively. In both devices, a progressive increase in temperature was observed over the first 40 min, after which the solution temperature stabilized and remained constant until the end of the counting time at 120 min. This increase in temperature over the first 40 min is due to heat released by the UV LED irradiation system (12W, 365 nm) that is transferred to the solution, gradually raising the temperature. It was observed that the Arduino Max6675 sensor recorded a temperature difference of 2–4 °C higher than that measured by the commercial K-29 thermometer at the beginning and throughout the experiment. It is important to mention that when assembling the MAX6675 sensor, the thermocouple tip was inserted inside a sealed glass cylinder to protect it from oxidation and facilitate the movement of the sensor inside the reservoir (Figure 2b). This temperature difference is related to the effect of thermal conduction present between the solution, the glass, and the metal of the thermocouple.^41^

**Figure 6:**
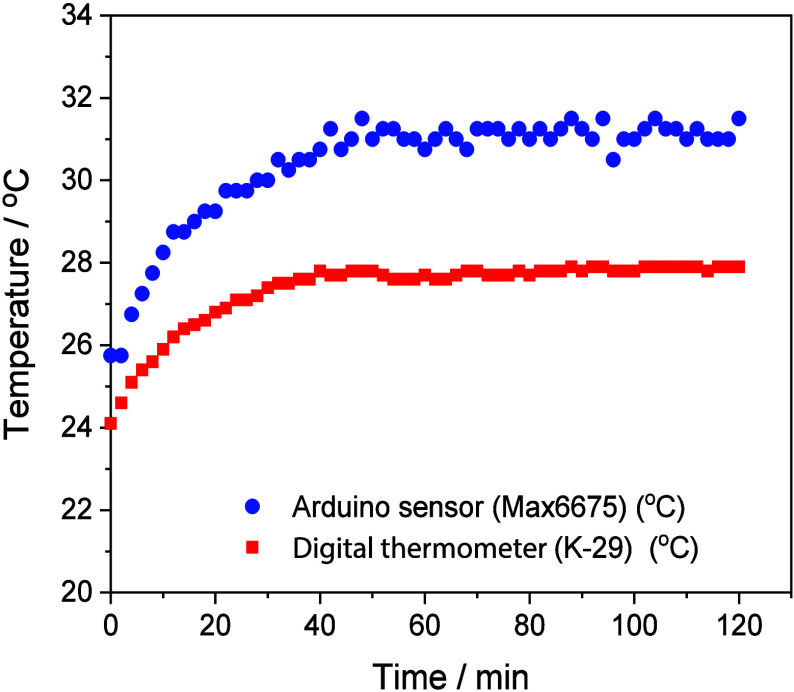
Performance and stability assessment of temperature sensors (Arduino sensor Max6675 and Digital thermometer K-29) (*n* = 1) in a recirculating loop. *Experimental conditions* **:** CF/WO_3_ membranes; pH 6.5; VEN concentration: 1.0 mg L^–1^; volume 150 mL; LED (365 nm, 12 W) and flow: 210 mL min^–1^.

Temperature control is considered to be a critical parameter in many industrial processes. In this context, Paduloh^42^ developed a device using the Max6675 and GSM SIM800L temperature sensors, combined with an Arduino UNO, for temperature monitoring and superheat control in an agitator for emulsions and synthetic resins. The device results, compared with a thermal gun, showed temperature differences of 1 and 2.5 °C, respectively. In addition, the device containing Max6675 provides fast, accurate temperature readings for extended periods, helping prevent significant agitator damage from rapid motor heating.

Septiana^43^ developed a simple system to measure the temperature in different fluids, using 4 type K thermocouples with MAX6675 modules connected to an Arduino microprocessor with DS18B20 and calibrated using an ASTM-117C thermometer. The system using MAX6675 sensors proved to be effective in monitoring this parameter in water samples, reaching an initial error of 4.9%, making it possible to reduce it to 0.4%, in addition, it demonstrated high precision, allowing rapid data acquisition (every 0.1 s) and enabling the storage of information in txt format.

#### 3.4.3 pH Sensor Evaluation

The pH sensor was evaluated under both static conditions, using a glass reservoir, and dynamic conditions in a recirculating loop incorporating a filtration/photocatalysis reactor (Figure 7). In static condition, standard buffer solutions (NEON brand) evaluated at pH 4.0; 7.0 and 10.0 at 25 °C were used (Figure 7a). The results showed similar behavior when studying pH 4.0 and 7.0 in both devices (Figure 7a), showing values of 3.93 ± 0.03 and 6.98 ± 0.01 when using the commercial pH meter Kasvi/K-39–2014B, as well as values of 3.92 ± 0.03 and 6.98 ± 0.03 obtained under the PH4502C sensor.

**Figure 7:**
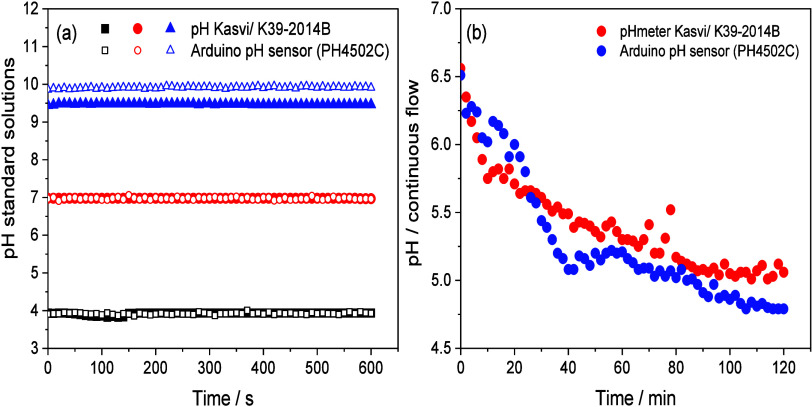
Performance and stability evaluation of pH sensors (pHmeter Kasvi/K39–2014B and Arduino pH sensor PH4502C) under (a) static conditions and (b) dynamic conditions in a recirculating loop. *Experimental conditions:* CF/WO_3_ membranes; pH (4.0, 7.0, and 10) (*n* = 1) in static conditions, pH 6.5 in dynamic conditions (*n* = 1); VEN concentration: 1.0 mg L^–1^; volume 150 mL; LED (365 nm, 12 W) and flow 210 mL min^–1^.

However, at pH 10.0, a difference of approximately 0.5 was observed between the commercial Kasvi pH meter (9.47 ± 0.01) and the Arduino sensor (9.91 ± 0.2). The %RSD for all data sets ranged from 0.11% to 0.70%.

During the pH evaluation in a recirculating loop (Figure 7b), both sensors recorded a decrease in pH throughout the experiment. The pH ranged from 6.5 to 5.1 using the commercial Kasvi pH meter, indicating a change of 1.4 after 120 min. With the Arduino PH4502C sensor, the variation was from 6.5 to 4.8, corresponding to a change of 1.7. For both devices, % RSD of 6.38% and 9.13% were obtained, respectively.

### 3.5 Statistical Analysis

Statistical tests at 95% confidence were performed to assess the variance between the OMSys and each of the commercial instruments (pH meter Kasvi/K39–2014B and digital thermometer K-29). Table 3 shows that when comparing the static pH of 4.0 and 7.0, p_(two‑tailed)_ 0.524 and 0.359 were observed, respectively > α 0.05, indicating that the null hypothesis is not rejected and that there is not enough evidence to say that the group means are statistically different. For pH 10, the opposite was observed with p­(two-tailed) 9.69 × 10^–104^ < α = 0.05. Since the p-value is less than α, we reject the null hypothesis, indicating that the group means differ, as noted in item 3.4.3.

**Table 3:** Statistical Determination of Variance and Test with 95% Confidence

	Variance			*t* test	
Measures	Degrees of freedom	pH KASVI K39-2014B	Arduino pH Sensor PH4502C	p_two‑tailed_	Observations
4.0 pH_estatic_	120/60 each	0.00097	0.00063	0.524 > α=0.05	No statistically significant difference between OMSys and the equipment
7.0 pH_estatic_		4.75 × 10^–6^	0.00084	0.359 > α=0.05	
10.0 pH_estatic_		0.00010	0.00047	9.69 × 10^–104^ < α=0.05	Are there differences between OMSys and the equipment
pH_dynamic_	240/120 each	0.119	0.233	0.118 > α=0.05	No statistically significant difference between OMSys and the equipment

Measures	Degrees of freedom	Digital Thermometer K-29	Arduino sensor MAX6675 chip	p_(two-tailed)_	Observations
Temperature	120/60 each	0.743	1.882	1.11 × 10^–26^ < α=0.05	Are there differences between OMSys and the equipment

When pH continued in the application of the treatment of water contaminated with the antidepressant venlafaxine, it was observed that after 240 degrees of freedom, p_(two‑tailed)_ 0.118 > α = 0.05, indicating that the null hypothesis is not rejected and that there is not enough evidence to say that the means of the groups are statistically different.

Comparing the commercial thermometer with the MAX6675 chip Sensor, a two-tailed p-value of 1.11 × 10^–26^ < α= 0.05 was observed. In this case, we need to reject the null hypothesis that the group averages are statistically different, as reported in the problems in item 3.4.2, variations related to the protection of the temperature sensor glass that cause heat transfer, making the sensor much less sensitive.

### 3.6 Application of OMSys to the Treatment of Contaminated Water

After the parameters flow, temperature, and pH were evaluated separately, the OMSys was finally applied to monitor these parameters in the treatment of water contaminated by the degradation of the antidepressant venlafaxine. The OMSys was coupled with a filtration/photocatalysis reactor operating in a recirculating loop using a CF/WO_3_ membrane and a UV LED irradiation system (365 nm-12 W).

The degradation of the antidepressant VEN was evaluated in different control experiments. Under direct photolysis (without a catalyst), a low degradation of only 9% (0.09 mg L^–1^) was observed. However, during the adsorption process (in the dark), the degradation increased by 21% (0.21 mg L^–1^), demonstrating that both direct photolysis and adsorption in the dark resulted in negligible degradation of the antidepressant after 120 min. When the process was evaluated using photocatalysis (photoactive membrane + light), 80% degradation (0.80 mg L^–1^) was observed after 120 min (Figure 8a).

**Figure 8:**
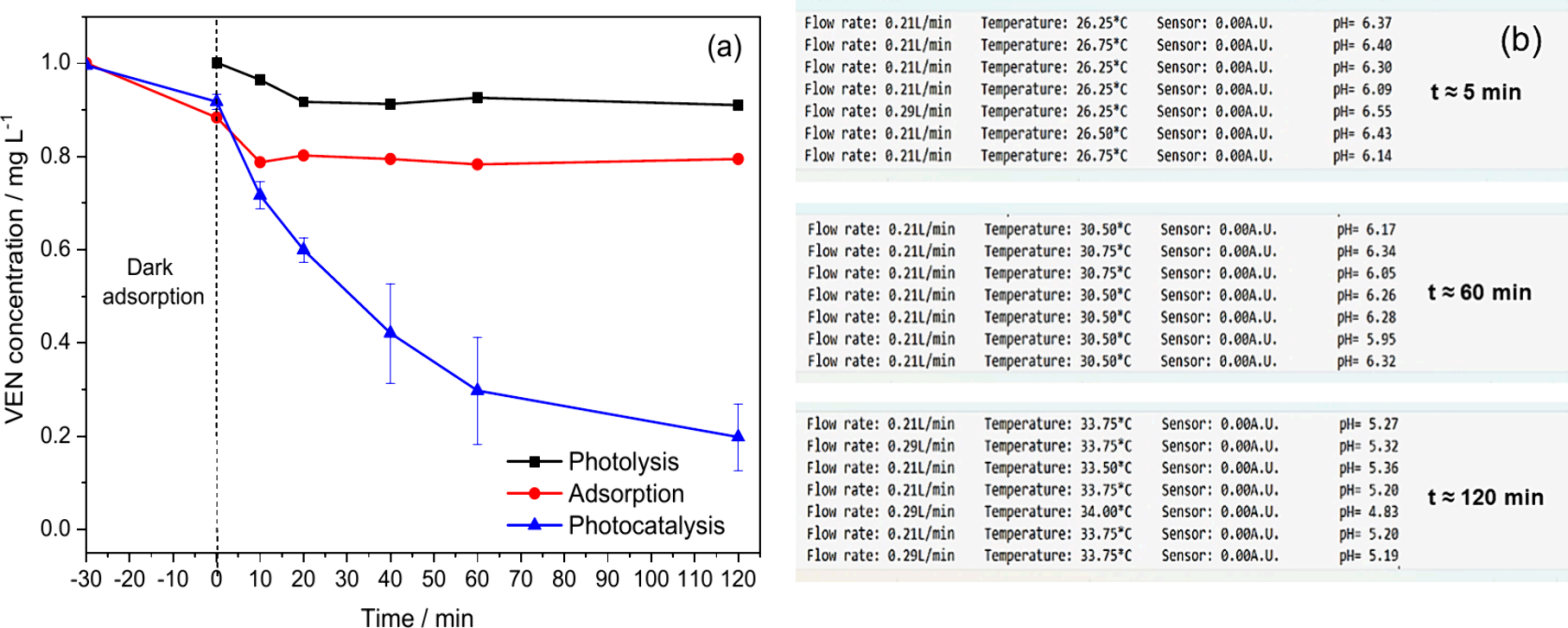
Evaluation of (a) photolysis (*n* = 1), adsorption (*n* = 1), and photocatalysis (*n* = 3) effect on venlafaxine degradation. (b) Monitoring of the parameters: flow, temperature, and pH at the times: 5, 60, and 120 min of the photocatalysis experiment. *Experimental conditions*: CF/WO_3_ membranes, pH: 6.5; VEN concentration: 1.0 mg L^–1^; volume: 150 mL, LED (365 nm), flow: 210 mL min^–1^.

The experiments of direct photolysis, adsorption in the dark, and photocatalysis showed increasing apparent kinetic constants (*k*_app_) of 0.0023, 0.0035, and 0.0091 ± 0.0012 min^–1^, respectively, for the degradation of VEN after 120 min, indicating clear differences in the mechanisms involved.

In photolysis, which showed the lowest *k*_app_ value, this suggests a low susceptibility of the drug VEN to photochemical degradation. Dark adsorption, which showed a slight increase in *k*_app_, suggests some interaction between VEN and the FC/WO_3_ membrane surface, allowing partial removal due to possible retention on the membrane surface. Finally, photocatalysis, which showed a considerable increase in the *k*_app_, confirms that the process occurs predominantly via oxidation.

This degradation of VEN is related to the broad absorption of the WO_3_ semiconductor in the emission region of the UV-LED irradiation system (365 nm).^44^ Furthermore, the deposition of WO_3_ on the surface of the carbon fiber decreases the recombination rate of the electron (e^–^)/ hole (h^+^) pairs that act as a heterojunction, facilitating the displacement of electrons across the surface of the carbon fiber, delaying their recombination, thus favoring catalytic activity.^29^ The reduction of recombination by electron displacement has already been observed in materials that have carbon in their composition, such as composites formed by graphene oxide sheets modified with tungsten oxide (GO/WO_3_).^45^

In turn, OMSys has proven to be an interesting tool when used in photocatalytic membrane filtration processes applied in the treatment of contaminated waters, as it enables continuous and uninterrupted data acquisition at 2.0 s intervals along with real-time data transmission via Wi-Fi, offering a more detailed view of the behavior of the filtration/photocatalysis process during the experiment.

In this context, the monitoring and observed variations of the parameters: temperature from 26 to 34 °C and pH from 6.5 to 5.1 after 2 h in the photocatalysis process, would be related, respectively, to the heat emission from the LED irradiation source and to the leaching of W in the solution, referring to material losses from the FC/WO_3_ membrane. These variations can be better visualized in Figure 8b, which shows the real-time monitoring of the three parameters (flow, temperature, and pH) at specific times (5, 60, and 120 min) throughout the photocatalysis experiment. Furthermore, the screenshots (Figure 8b) illustrate the progressive decrease in pH over time during the photocatalysis experiment.

A significant limitation of the pH sensor (PH4502C) is that it exhibited pronounced oscillations in the measurements (Figure 8b), related to its position in the reservoir (near the power inlet), which generated disturbances that altered the electrochemical equilibrium at the electrode-medium interface, producing variations in the measured potential. Furthermore, the PH4502C sensor exhibits greater sensitivity to electrical noise and to temporal variations in the solution.^46^

During the photocatalysis experiment (Figure 8a), the gradual decrease in pH throughout the experiment was attributed to the presence of tungsten (W) nanoparticles in the solution, associated with leaching or loss of the electrodeposited material on the membrane.^29,47^ To confirm this hypothesis, the elemental quantification of tungsten (W) was analyzed by ICP-OES at the initial (t = 0 min) and final (t = 120 min) stage of the experiment (Figure 9a), yielding values of < LOD (0.015 mg L^–1^) and 2.51 ± 0.0903 mg L^–1^, respectively.

**Figure 9:**
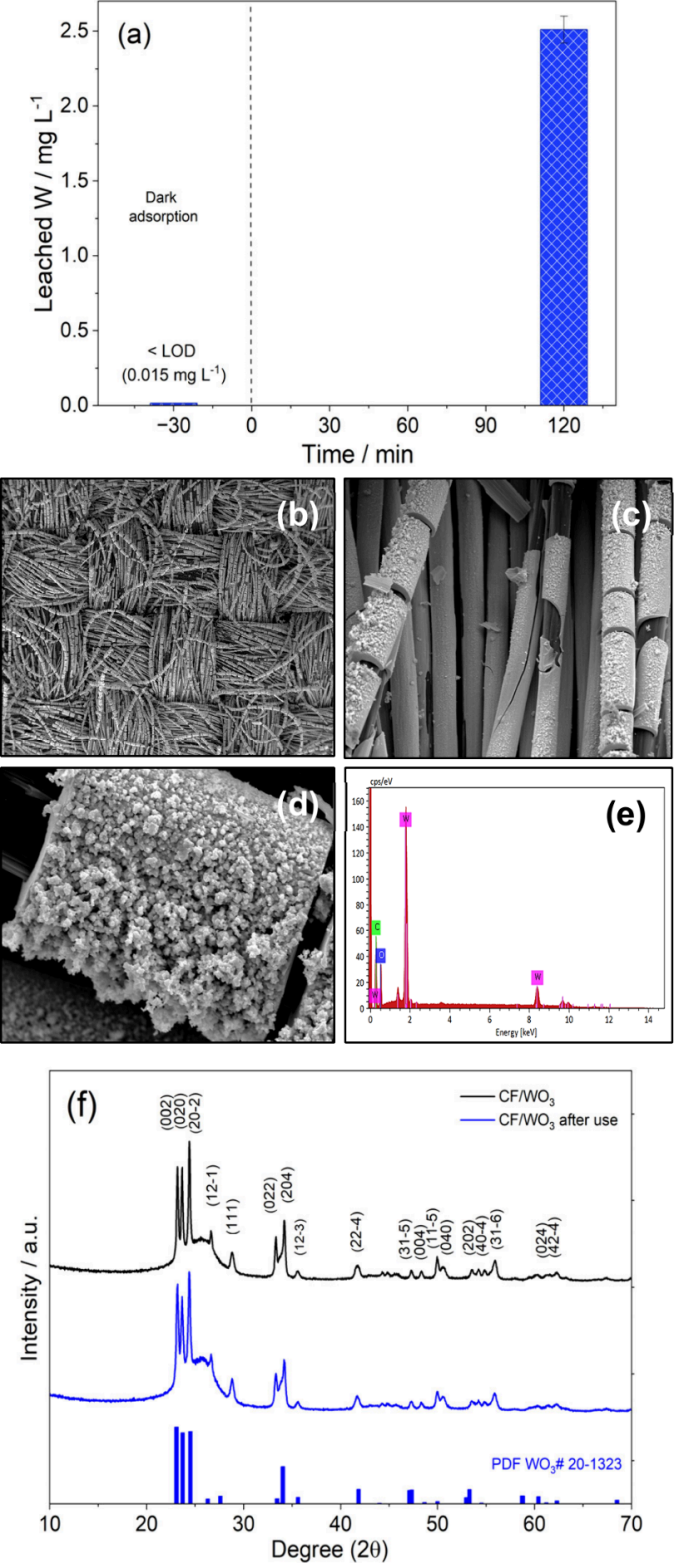
(a) Determination of W in solution at the initial and final stage of the photocatalysis experiment using ICP-OES (*n* = 3). FEG-SEM images for CF/WO_3_ membrane after use (photocatalysis experiment) in (b) magnification 50X, (c) magnification 300X, and (d) magnification 3300X. (e) Energy dispersive X-ray analysis (EDX) and (f) X-ray diffractograms of CF/WO_3_ membranes before and after the photocatalysis experiment.

Catalyst leaching during photocatalytic experiments, especially in systems using semiconductor-modified membranes, is widely known.^48^ When it comes to a filtration-photocatalysis process in which the fluid is forced to pass through a CF membrane modified with WO_3_ on its surface, it is normal for part of the semiconductor to fragment and be transferred to the solution. In this context, the leaching of species such as W contributes to the acidification of the solution.

This progressive increase in acidity affects the efficiency of the photocatalytic process.^26^ For this same membrane under similar study conditions, W leaching was observed.^29^ To verify this situation, FEG-SEM micrographs of the membranes after use were taken (Figure 9b-d). The results confirmed partial fragmentation of the semiconductor on the surface of the carbon fiber membrane (PWB3), indicative of WO_3_ nanoparticles, which explained the change in solution pH. On the other hand, EDX analysis after use of the membrane (Figure 9e) confirmed the presence of the elements C, W, and O.

The X-ray diffraction (XRD) pattern shown by the membrane under conditions before and after the photocatalysis process (Figure 9f) was compared with the Powder Diffraction File (PDF) (no. 20–1323), showing the following characteristic peaks for monoclinic WO_3_ with crystalline planes at: 2θ: 23.1° (0 0 2), 23.3° (0 2 0), 23.5° (20–2), 26.3° (12–1), 28.8° (1 1 1), 33.0° (0 2 2), 33.4° (20–4), 35.2° (12–3), 41.8° (22–4), 44.2° (31–5), 45.2° (0 0 4), 47.3° (0 4 0), 48.4° (11–5), 50.5° (40–4), 53.1° (2 0 2), 53.5° (31–6), 54.8° (0 2 4), 55.9° (42–4), 60.3° (42–6), 61.3° (34–3), and 62.3° (31–7).

When comparing both diffractograms (Figure 9f), no phase transformations or variations in the intensity and position (2θ) of the main crystalline peaks of WO_3_ were observed, confirming that there were no changes in the crystallinity of the membrane.

It is known that pH variation affects photocatalytic efficiency, as it directly influences surface properties.^49^ This leaching effect has already been reported in filtration/photocatalysis processes using semiconductor-modified membranes, Ghalamchi^50^ with Ag_3_PO_4_/CuZnAl-NLDH, showed Ag release rates between 0.038% and 4.2%. In this context, Wang,^51^ developed PVDF/CdS/Bi_2_WO_6_/ZnO membranes for the degradation of nitrite (NO_2_) in water. The results showed a degradation efficiency greater than 92% of nitrite after 4 h under visible light, its influence being related to the effects of pH and demonstrating that a decrease in pH to 4.0 promoted by the increase in the H^+^ content in the solution (as well as by the protonation of the membrane surface favoring the occurrence of electrostatic interactions between the anionic NO_2_ and the protonation sites, which finally allows an increase in the photocatalytic activity and greater degradation of NO_2_. However, an increase in pH to> 7.0 resulted in a significant reduction in photocatalytic response.

The variations shown by the parameters allow for the identification of problems in situ, facilitating rapid decision-making, minimizing failures, and reducing operational costs by avoiding unnecessary experiments, reagent expenses, or the use of new membranes. This is ideal in photocatalytic processes, as pH changes can be detected and corrected while maintaining ideal process conditions. Similarly, in filtration systems, abrupt variations in flow identify problems associated with fouling, deformation, or possible membrane rupture, facilitating maintenance.

The OMSys demonstrated proper functioning in monitoring all parameters (flow, temperature, and pH), allowing for use over periods (>2 h). Its portability facilitated successful integration into filtration/photocatalysis reactor equipment, but it can also be used in other devices (cells or tanks), enabling field monitoring and eliminating the need for complex structures and costs. As a system based on an ESP32 microcontroller (240 MHz, 64 MB flash memory) with Wi-Fi and Bluetooth connectivity, it enables rapid, real-time data storage. As an open-source platform, it is reprogrammable and customizable. Its low cost, proven application in contaminant handling processes, and scalability potential make it viable for use in laboratories.

To understand OMSys’s innovation of OMSys relative to the current state of the art, mention some related systems (Table 4). In general, some of these referenced systems focus on the isolated acquisition of parameters, have low sampling rates (min), lack actuator actuation or control in reactors or similar equipment, and do not evaluate hybrid processes such as filtration/photocatalysis.

**Table 4:** Comparative Analysis of the OMSys and Previously Reported Monitoring Systems

Parameters evaluated	Sampling rate	Data transmission	Actuator control	Aplication	ref.
pH, temperature, turbidity, conductivity	Not specified	Wi-Fi ESP32	No	Water quality	^52^
Temperature	min	ESP32 and sensor DS18B20	No	Aquaculture	^53^
Condutivity Temperature	s/min	Wi-Fi (ESP32)	No	Ultrapure water/environmental monitoring	^54^
Flow rate, pH Temperature (simultaneous)	(2.0 s) High frequency	WiFi/Bluetooth + (automatic storage)	Yes (LED light source and peristaltic pump)	Filtration/photocatalysis system (VEN degradation)	OMSys (present study)

In contrast, OMSys avoids these limitations by integrating real-time multiparameter monitoring, at-will actuation of actuators such as LED irradiation sources and peristaltic pumps complementary to the system, automatic storage, fast data acquisition (2.0 s), enabling rapid decision-making in the face of critical events such as pH correction, and finally, evaluation of its practical application in processes such as the real degradation of emerging contaminants, absent in the systems referenced in Table 4.

Although there are few studies in the specialized literature presenting a specific device for monitoring physical-chemical parameters applied to hybrid filtration/photocatalysis processes, several studies with similar configurations applied in the evaluation of water quality are mentioned^55,56^ or articulated in equipment in photocatalytic reactors.^57^ In this context, Hong^58^ developed a device with multiple sensors using the Arduino Uno R3 board as a microcontroller to analyze water quality by monitoring pH, temperature, turbidity, and total dissolved solids (TDS). Although the system proved to be functional and effective in monitoring the parameters, achieving standard deviation (RDS) for temperature (0.98); Turbidity (444.56), TDS (188.75), and pH (5.17), values close to those obtained using our OMSys device, showed certain disadvantages, such as limited storage capacity, in addition to the absence of data transmission systems via Wi-Fi or Bluetooth, limiting the analysis of results in real time, especially for long experiments, even requiring the presence of an operator. These problems can be solved by using more advanced microcontrollers (ESP32, Arduino Uno R4) that offer greater processing power, expanded memory, and connectivity (Wi-Fi and Bluetooth), enabling quick connections to networks or other devices.

The use of ESP32 in conjunction with commercial sensors has demonstrated significant advantages in equipment design. Kalamaras^6^ developed a low-cost device based on an ESP32 microcontroller, combined with commercial sensors, to monitor the parameters pH, temperature, Redox potential, and ammonium concentration, coupled to an anaerobic digestion bioreactor. As it is an ESP32-based device, data recording and storage occur effectively. The possibility of reprogramming ESP32 with free software such as the Arduino IDE, along with sensor-specific libraries, enables the development of an efficient system and reduces operational costs. Similarly, the developed OMSys achieved efficiency levels comparable to those reported by Kalamaras,^6^ while maintaining the advantages associated with the use of ESP32 and proving to be an affordable alternative for experimental reactor applications.

Therefore, it can be highlighted that prototyping systems based on microcontrollers such as ESP32 can be considered a smart choice for the development of portable and low-cost devices aimed at monitoring fundamental parameters such as pH, temperature, and flow, due to their outstanding versatility allowing integration with sensors or various electrical components, proper real-time data processing, easy communication, and data capture, along with low energy consumption and the possibility of implementation in external installations or systems, make them a valuable alternative for effective control and management in hybrid filtration/photocatalysis processes associated with water treatment.

## 4 Conclusions

From a technological perspective, the OMSys system represents a significant advancement in environmental monitoring and analytical instrumentation and can be considered an effective and interesting tool for the in situ analysis of real matrices and for industrial process applications. The system offers important advantages, including rapid acquisition, high processing capacity, and automated, real-time data storage for monitoring physicochemical parameters such as pH, temperature, and flow during filtration/photocatalysis processes used to treat contaminated water. In addition, OMSys met the requirements for data reliability and efficiency, improving experimental logistics in degradation studies and yielding relative standard deviation (RSD) values between 0.1% and approximately 10% under the best-evaluated conditions, a range recognized in the literature as indicative of analytical precision.

The system also demonstrated a high capacity to monitor, in real time, small variations in parameters critical to the degradation of the antidepressant venlafaxine, while its simple and intuitive interface allows for efficient process control, facilitating operational adjustments, such as pH corrections and the identification of temperature or flow variations. Continuous reading, combined with automatic data storage, contributed to greater analytical reliability, validating the role of OMSys as a promising tool for optimizing studies of recirculating loop reactors and electrochemical cells. Additionally, the possibility of integrating new sensors (conductivity and turbidity) expands accessibility, while its portability and compatibility with smartphones and computers reduce operational costs and eliminate the need for constant on-site monitoring, reinforcing its versatility and applicability across diverse experimental and environmental scenarios.

## Supplementary Material



## Notes

A detailed description of the complete source code for OMSys and FFT-Based Low-Pass Filtering (0.0625 min^–1^ Cutoff) for Volumetric Flow Rate Sensor Signal Processing is available in Appendix A and Figure S1 of the Supporting Information, respectively (PDF)
