# Supplementary material for: Integration of Technologies for Advanced Monitoring and Improved Performance of Filtration/Photocatalysis Systems in Water Treatment
Source: ACS Omega. 2026 Apr 10;11(15):23263–76. doi: 10.1021/acsomega.6c00220 (PMC13103826; doi:10.1021/acsomega.6c00220)

Supplementary Information

Integration of technologies for advanced monitoring and improved performance of filtration/photocatalysis systems in water treatment.

Hernán Dario Rojas-Mantilla*†; Juliano Passaretti Filho†; Saidy Cristina Ayala-Durán; Maria Valnice Boldrin Zanoni.

São Paulo State University (UNESP), Institute of Chemistry, Department of Analytical Chemistry, National Institute of Alternative Technologies for the Detection, Toxicological Evaluation and Removal of Micropollutants and Radioactivies (INCT-DATREM), Rua Professor Francisco Degni, 55, Araraquara - 14800-060, São Paulo State, Brazil.

†These authors contributed equally to this work.

*Corresponding author: Hernán Dario Rojas Mantilla

Address: Rua Prof. Francisco Degni 55, Araraquara, SP, 14800-060, Brazil

Phone: +55 16 3301 9519

E-mail: hernan.mantilla@unesp.br

Appendix A

```
//* Definições: GPIOs MAX6675 */https://github.com/adafruit/MAX6675-library
#include "max6675.h"
uint8_t SO = 18;
uint8_t CS = 19;
uint8_t CLK = 21;
MAX6675 sensor(CLK, CS, SO);
float Temperature;

const int output26 = 26;
const int output27 = 27;


int analogPin = 35;
int val = 0;

//* https://how2electronics.com/iot-water-flow-meter-using-esp8266-water-flow-sensor/
#define SENSOR  4

long currentMillis = 0;
long previousMillis = 0;
int interval = 1000;
boolean ledState = LOW;
float calibrationFactor = 4.5;
volatile byte pulseCount;
byte pulse1Sec = 0;
float flowRate;
unsigned long flowMilliLitres;
unsigned int totalMilliLitres;
float flowLitres;
float totalLitres;

void IRAM_ATTR pulseCounter()
{
  pulseCount++;
}


// Load Wi-Fi library
#include <WiFi.h>

// Replace with your network credentials
const char* ssid = "YOURNETWORK";
const char* password = "YOURPASSWORD";

// Set web server port number to 80
WiFiServer server(80);

// Variable to store the HTTP request
String header;

// Auxiliar variables to store the current output state
String output26State = "on";
String output27State = "on";

// Current time
unsigned long currentTime = millis();
// Previous time
unsigned long previousTime = 0;
```

```
// Define timeout time in milliseconds (example: 2000ms = 2s)
const long timeoutTime = 2000;


void setup() {
  Serial.begin(115200);
  // Initialize the output variables as outputs
  pinMode(output26, OUTPUT);
  pinMode(output27, OUTPUT);
  // Set outputs to HIGH
  digitalWrite(output26, HIGH);
  digitalWrite(output27, HIGH);


  // Connect to Wi-Fi network with SSID and password
  Serial.print("Connecting to ");
  Serial.println(ssid);
  WiFi.begin(ssid, password);
  while (WiFi.status() != WL_CONNECTED) {
    delay(500);
    Serial.print(".");
  }
  // Print local IP address and start web server
  Serial.println("");
  Serial.println("WiFi connected.");
  Serial.println("IP address: ");
  Serial.println(WiFi.localIP());
  server.begin();
  pulseCount = 0;
  flowRate = 0.0;
  flowMilliLitres = 0;
  totalMilliLitres = 0;
  previousMillis = 0;
  attachInterrupt(digitalPinToInterrupt(SENSOR), pulseCounter, FALLING);

}

void loop(){
  Temperature = (sensor.readCelsius());
  val = analogRead(analogPin);
  currentMillis = millis();
  if (currentMillis - previousMillis > interval)
  {
    pulse1Sec = pulseCount;
    pulseCount = 0;

    flowRate = ((1000.0 / (millis() - previousMillis)) * pulse1Sec) / calibrationFactor;
    previousMillis = millis();

    flowMilliLitres = (flowRate / 60) * 1000;
    flowLitres = (flowRate / 60);

    totalMilliLitres += flowMilliLitres;
    totalLitres += flowLitres;

    Serial.print("Flow rate: ");
    Serial.print(float(flowRate));
    Serial.print("L/min");
    Serial.println("\t");
    Serial.print("Temperatura: ");
```

```
    Serial.print(sensor.readCelsius());
    Serial.print("*C");
    Serial.println("\t");
    Serial.print("Condutividade: ");
    Serial.print(float(val));
    Serial.print("U.A.");
    Serial.println("\t");

}


  WiFiClient client = server.available();   // Listen for incoming clients

  if (client) {                             // If a new client connects,
    currentTime = millis();
    previousTime = currentTime;
    Serial.println("New Client.");          // print a message out in the serial port
    String currentLine = "";                // make a String to hold incoming data from the client
    while (client.connected() && currentTime - previousTime <= timeoutTime) {  // loop while the
client's connected
      currentTime = millis();
      if (client.available()) {             // if there's bytes to read from the client,
        char c = client.read();             // read a byte, then
        Serial.write(c);                    // print it out the serial monitor
        header += c;
        if (c == '\n') {                    // if the byte is a newline character
          // if the current line is blank, you got two newline characters in a row.
          // that's the end of the client HTTP request, so send a response:
          if (currentLine.length() == 0) {
            // HTTP headers always start with a response code (e.g. HTTP/1.1 200 OK)
            // and a content-type so the client knows what's coming, then a blank line:
            client.println("HTTP/1.1 200 OK");
            client.println("Content-type:text/html");
            client.println("Connection: close");
            client.println();


            // turns the GPIOs on and off
            if (header.indexOf("GET /26/on") >= 0) {
              Serial.println("GPIO 26 off");
              output26State = "on";
              digitalWrite(output26, HIGH);
            } else if (header.indexOf("GET /26/off") >= 0) {
              Serial.println("GPIO 26 on");
              output26State = "off";
              digitalWrite(output26, LOW);
            } else if (header.indexOf("GET /27/on") >= 0) {
              Serial.println("GPIO 27 off");
              output27State = "on";
              digitalWrite(output27, HIGH);
            } else if (header.indexOf("GET /27/off") >= 0) {
              Serial.println("GPIO 27 on");
              output27State = "off";
              digitalWrite(output27, LOW);
            }



            // Display the HTML web page
            client.println("<!DOCTYPE html><html>");
```

```
        client.println("<head><meta name=\"viewport\" content=\"width=device-width, initial-
scale=1\">");
        client.println("<link rel=\"icon\" href=\"data:,\">");
        // CSS to style the on/off buttons
        // Feel free to change the background-color and font-size attributes to fit your
preferences
        client.println("<style>html { font-family: Helvetica; display: inline-block; margin: 0px auto;
text-align: center;}");
        client.println(".button { background-color: #4CAF50; border: none; color: white; padding:
16px 40px;");
        client.println("text-decoration: none; font-size: 30px; margin: 2px; cursor: pointer;}");
        client.println(".button2 {background-color: #555555;}</style></head>");


        // Web Page Heading
        client.println("<body><h1>Controle fotoreator</h1>");

        // Display Termopar
        client.println("<tr><td>Temp. Celsius</td><td><span class=\"sensor\">");
        client.println(sensor.readCelsius());
        client.println(" *C</span></td></tr>");

        // Display condutimetro
        client.println("<tr><td>Condutividade</td><td><span class=\"sensor\">");
        client.println(val);
        client.println(" U.A.</span></td></tr>");

        // vazao
        client.println("<tr><td>Vazao</td><td><span class=\"sensor\">");
        client.println(float(flowRate));
        client.println(" Lmin-1</span></td></tr>");

        // Display current state, and ON/OFF buttons for GPIO 26
        client.println("<p>GPIO 26 - State " + output26State + "</p>");
        // If the output26State is off, it displays the ON button
        if (output26State=="off") {
          client.println("<p><a href=\"/26/on\"><button class=\"button\">ON</button></a></p>");
        } else {
          client.println("<p><a href=\"/26/off\"><button class=\"button
button2\">OFF</button></a></p>");
        }

        // Display current state, and ON/OFF buttons for GPIO 27
        client.println("<p>GPIO 27 - State " + output27State + "</p>");
        // If the output27State is off, it displays the ON button
        if (output27State=="off") {
          client.println("<p><a href=\"/27/on\"><button class=\"button\">ON</button></a></p>");
        } else {
          client.println("<p><a href=\"/27/off\"><button class=\"button
button2\">OFF</button></a></p>");
        }
        client.println("</body></html>");

        // The HTTP response ends with another blank line
        client.println();
        // Break out of the while loop
        break;
      } else { // if you got a newline, then clear currentLine
        currentLine = "";
      }
```

```
      } else if (c != '\r') {  // if you got anything else but a carriage return character,
        currentLine += c;      // add it to the end of the currentLine
      }
    }
  }
  // Clear the header variable
  header = "";
  // Close the connection
  client.stop();
  Serial.println("Client disconnected.");
  Serial.println("");
}
}
```

Figure S1- FFT-Based Low-Pass Filtering (0.0625 min^{-1} Cutoff) for Volumetric Flow Rate Sensor Signal Processing.

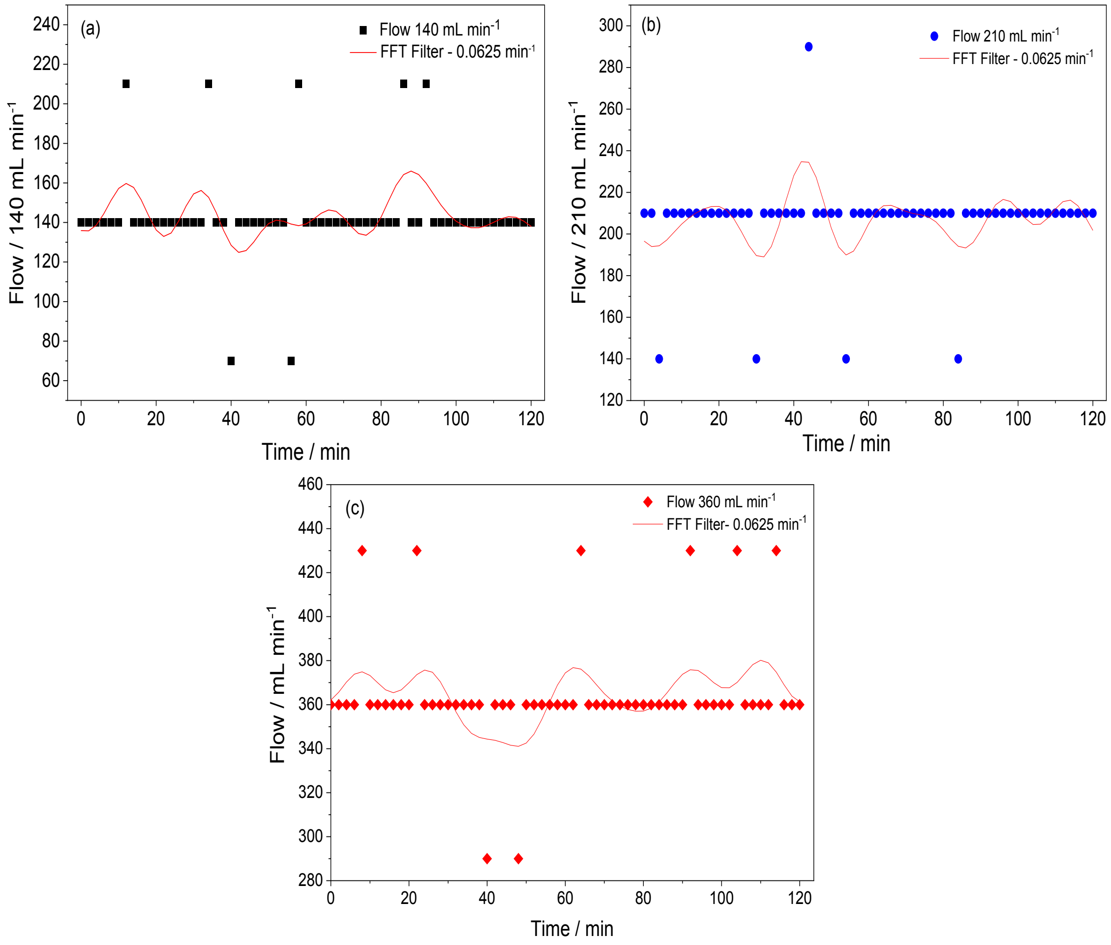

Supplement: Supplementary file 1 [file ao6c00220_si_001.pdf]
